# Index to Authors

**Published:** 1933

**Authors:** 


### INDEX TO AUTHORS

**Ackland, J. M.**, Facial neuralgia and dental irritation, **4**, 28.

**Ackland, W. R.**, Effect of general conditions on the quality of the teeth, **34**, 118. Oral sepsis as a source of systemic infections, **39**, 76. Grave cardiac symptoms due to multiple dental abscess, **41**, 79.

**Adams, A. W.**, Spinal and peripheral nerve injuries, **42**, 32. Congenital club foot, **43**, 232. Pyelography in renal disorders, **46**, 49. Ponto-cerebellar tumour, **49**, 309. *See also* **Birrell, J. A.**

**Alexander, D. A.**, Abdominal fibroma simulating splenomegaly, **26**, 60. Cretinism and the puerperium **27**, 128. Raynaud's disease **30**, 247.

**Alford, H.**, The Bristol Infirmary in my student days, 1822–1828, **8**, 165.

**Allbutt, T. C.**, Prevention of apoplexy, **23**, 1.

**Almond, G. H. H.**, Pulmonary percussion notes, **27**, 215. Oxycephaly, **28**, 118. Urinary products of intestinal intoxication, **31**, 130. Clinical examination of stools, **33**, 95.

**Ashton, L. P.**, Von Recklinghausen's disease, **47**, 219.

**Aubrey, T., and Britton, R. B.**, An epidemic of virulent diphtheria, **46**, 141.

**Bannatyne, G. A.**, Epilepsy treated by sulphonal, **9**, 261.

**Bardswell, N.**, Mixed infections in pulmonary tuberculosis, **32**, 125.

**Barker, A. E.**, Thrombosis of femoral artery, **11**, 84.

**Baron, B. J.**, Excoriationes narium, **4**, 52. Laryngoscope in medicine, **4**, 145. Phthisical hoarseness and aphonia, **6**, 12. Laryngeal growths, **7**, 78. Outgrowths on tongue, **8**, 80. Chronic suppuration of the maxillary antrum, **15**, 13. Paralysis of right vocal cord due to thoracic aneurysm, **19**, 208. **P.A.** Progress in treatment of throat, nose and ear disease during the past twenty years, **19**, 289. *See also* **Skerritt, E. M.**

**Beaumont, W. M.**, Ptosis, **20**, 131. Paralysis of accommodation, **20**, 213.

**Beddoe, J.**, Address to Bristol Medical School, **1**, 235. Clifton, **7**, 114. **L.F.** The first Long Fox Lecture, **22**, 303.

**Begg, C.**, Sprue, **23**, 138.

**Benham, H.**, Calculus on foreign body in bladder, **4,** 38.

**Berry, R. J. A.**, Hospitals—voluntary or self-supporting ? **47,** 19. Child guidance from the brain physiologist's point of view, **47,** 120. Mental deficiency, **49,** 177.

**Birrell, J. A.**, Post-influenzal tachycardia, **37,** 122. Acute suffocative œdema of the lungs, **44,** 239. Suprarenal virilism, **49,** 119. With **Adams, A. W.**, Congenital obstruction of the bile-duct, **48,** 215.

**Blachford, J. V.**, Recommendations of royal commission on lunacy, **44,** 15. Early diagnosis and treatment of insanity, **45,** 253. Epilepsy, **47,** 169.

**Blomfield, A. G.**, Landry's paralysis, **8,** 113. *See also* **Davy, H.**

**Bodman, F. H., and Bodman, P.**, Perforation in paratyphoid B., **47,** 317.

**Bodman, F. H., and Taylor, A. L.**, Portal pyæmia following diverticulitis, **46,** 131.

**Bodman, J. H.**, *see* **Rogers, B.**

**Boys, A. H.**, Priapism, **1,** 224.

**Brabazon, A. B.**, Bath, **10,** 254.

**Braine-Hartnell, C.**, Sanatoria for tuberculosis, **19,** 2.

**Brasher, C. W. J.**, Division of vasa deferentia for prostatic hypertrophy, **14,** 150. Alternating oxaluria and phosphaturia, **31,** 21. Intermittent swelling of parotid, **31,** 238. Hyperpiesis and arterio-sclerosis, **32,** 41.

**Brassington, H. W.**, Female circumcision amongst the Ameru, **49,** 237.

**Bristowe, H. C.**, Sterilization of the unfit, **48,** 189.

**Britton, R. B.**, *see* **Aubrey, T.**

**Brock, L. G.**, Modern outlook on mental health, **48,** 97.

**Brown, G. A.**, Bronchial croup, **4,** 115.

**Buckmaster, G. A.**, Applications of physiology to medicine, **37,** 7. Reaction of liquids of the body to indicators, **40,** 175. **L.F.** Cells and liquids of the body, **44,** 154.

**Bullen, F. St. J.**, Insanity, **30,** 205.

**Burder, G. F.**, **P.A.** Etiology of catarrh, **2,** 217.

**Bush, G. B.**, Redundant colon, **45,** 181. Silicosis—radiology and pathology, **48,** 259.

**Bush, J. P.**, Ligature of main arteries, **2,** 110. Rupture of aorta, **3,** 129. Cystotomy for bladder tumours of doubtful malignancy, **13,** 8. Unusual operations on lunatics, **17,** 219. Gastro-jejunostomy for cancer of pylorus, **20,** 57. Treatment of deformities by injection of paraffin, **20,** 220. **P.A.** Some surprises in my professional career, **21,** 289. Early history of Bristol Royal Infirmary, **26,** 193. With **Nixon, J. A.,** Filariasis, **25,** 218. With **Shaw, J. E.,** Sarcomatous tumour of shoulder-centre in cortex of right hemisphere, **13,** 99. *See also* **Shaw, J. E.**

**Bushnell, F.,** Thot ; an exalted surgeon and physician, **16,** 123. Antibodies in disease, **21,** 221.

**Cantlie, J.,** Effects of certain anatomical relations, **2,** 21.

**Carleton, H. H.,** Physiological conception of prolonged hysterical conditions, **39,** 36. Problems in pulmonary disease, **42,** 211 ; **43,** 54. Treatment of hæmoptysis, **45,** 39. Significance of sleep disorders, **46,** 173. Disorders of movement and their mechanism, **48,** 135. Trigeminal neuralgia, **49,** 47.

**Carling, E. R.,** Surgery for pain, **50,** 81.

**Carter, T. M.,** Dilatation of posterior cerebral vessels, with basal hæmorrhage in girl of sixteen, **26,** 322. With **Edgeworth, F. H.,** Morphia in uræmia, **20,** 231.

**Carwardine, T.,** Dermoid cyst of branchial cleft, **14,** 145. Pharyngeal pouch of Rathke, **15,** 310. Abdominal incisions, **18,** 204. Cystoscopy and ureteral catheterization, **19,** 303. Appendicitis, **20,** 319. Dangers of delay in ovariotomy, **21,** 309. Nephropexy by application of carbolic acid, **23,** 24. Notes on cases, **26,** 317. Indications for gastro-enterostomy, **27,** 107. Pericolitis, **31,** 333. Diagnosis of peptic ulcer, **40,** 71. Duodenal ulcers, **41,** 16 ; **45,** 279. **P.A.** Continuity and change, **42,** 201.

**Chambers, E. R.,** Ocular signs of migraine, **43,** 29. Early differential diagnosis of primary iritis, **48,** 51. Common ocular affections, **49,** 131.

**Charles, J. R.,** Effects of fright, **24,** 23. Treatment of diabetes with secretin, **24,** 218. Infection of the urinary tract with bacillus coli, **28,** 23. Epidemic cerebro-spinal meningitis, **31,** 142. Asthma, **42,** 84. Relation of autonomic nervous system to clinical medicine, **43,** 127. **P.A.** Relationship between structure and function, **49,** 1. Suprarenal hypertrophy, **49,** 115.

**Chiene, J.,** Mobility : sprains, dislocations and fractures, **21,** 97.

**Chitty, H.,** Artificial pneumothorax in phthisis, **30,** 141. Intussusception, **45,** 52. Seven hundred cases of acute appendicitis, **48,** 167. Anterior poliomyelitis, or infantile paralysis, **50,** 37.

**Christofferson, P. E.,** Swedish medical gymnastics in chronic heart disease, **18,** 134.

**Chudleigh, R. A.**, Method of expressing ear-power, **3**, 159.

**Clarke, C.**, Renal efficiency, **39**, 13. Diet and hygiene at the North Pole, **40**, 52.

**Clarke, J. Michell**, Graves's disease, **5**, 17. Peripheral neuritis, **5**, 73. Aneurysm of aorta, **6**, 130. Spinal cases, **6**, 120. Diseases of the heart, **6**, 126. Adult chorea, **6**, 131. Progressive muscular atrophy, **6**, 195. Dilatation of the stomach, **7**, 21. Tubercle of medulla oblongata, **7**, 194. Myxœdema, **7**, 258. Congenital heart disease, **7**, 261. Disseminated sclerosis, **7**, 265. Paraplegia, **8**, 226. Diagnosis in gastric affections, **9**, 73. Somnambulism, **12**, 81. Diagnosis of cancer and of simple dilatation of the stomach, **13**, 181. Oxygen in anæmia, **14**, 232. Acute miliary tuberculosis, **15**, 148. Enteric fever, **16**, 15. Temperature in apoplexy, **17**, 97. Sporadic cerebro-spinal meningitis, **18**, 128. Localized necrosis of lung, **20**, 122. Banti's disease, **21**, 14. Cerebellar tumours, **23**, 97. Relation of medicine to the natural sciences, **23**, 193. Myasthenia gravis, **23**, 308. Unusual complications of pneumonia, **25**, 89, 108. Treatment of Graves's disease by anti-thyroid serum and by X-rays, **25**, 201. Renal changes in a case of hæmorrhage into the pons, **26**, 230. **P.A.** Recent work on leukæmia and allied affections, **26**, 289. Precautions to be observed in the administration of milk in disease, **27**, 57. Leukæmia treated by X-rays, **28**, 208. **L.F.** Cerebro-spinal syphilis, **29**, 1. Chronic interstitial pancreatitis, **30**, 25. Acute septicæmia due to B. pyocyaneus, **31**, 4. Pernicious anæmia in older persons, **31**, 97. Diseases of lungs with Friedländer's bacillus, **32**, 1. Toxic polyneuritis of motor type, **33**, 9. Neuroses of the War, **34**, 89. Gunshot wounds of peripheral nerves, **35**, 57. With **Firth, J. L.**, Intra-cranial complications of ear disease, **30**, 97. With **Morton, C. A.**, Abscess in left lobe of cerebellum, **19**, 112.

**Clarke, R. C.**, The teething myth, **38**, 28. Medical treatment of duodenal ulcer, **45**, 289.

**Clarke, W. M.**, Intestinal obstruction, **3**, 38.

**Cobbold, C. S. W.**, Alleged increase of insanity, **11**, 145.

**Cole, T.**, Pernicious anæmia, **4**, 231.

**Connellan, P. S.**, War surgery in an Indian general hospital in Mesopotamia, **35**, 113. Treatment of repressed memories by hypnotism, **43**, 209.

**Cooke, R. V.**, Five cases of injury to main vessels occurring in civil practice, **47**, 311.

**Coombs, Carey F.**, Colitis polyposa, **24**, 31. Rheumatic carditis in childhood, **25**, 193. Causation of certain mid-diastolic murmurs, **26**, 312. Diffuse destructive changes in the liver associated with jaundice, **28,** 17. Chorea, **29,** 51. Cardiac arrhythmia, **29,** 321. Stokes-Adams syndrome, **31,** 30. The simulation of aortic aneurysm by other aortic and cardiac diseases, **32,** 26. Cardiac diseases and disorders in warfare, **33,** 149. Medicine and surgery in Mesopotamia, **34,** 136. **L.F.** Ætiology of cardiac disease, **43,** 1. Coronary obstruction, **44,** 249. Distal phenomena that accompany high arterial tension, **46,** 35. Work of the University Centre of cardiac research, **48,** 170. Coronary thrombosis, **49,** 277. Thirty years' progress in the study of rheumatic heart disease, **50,** 93. With **Herapath, C. E. K.,** Auricular failure, **41,** 180. *See also* **Stack, E. H. E.**

**Corfield C.,** What is an accident ? **31,** 148.

**Costobadie, H. P.,** Hypnotism, **28,** 33.

**Cotton, W.,** Simple form of influence machine for X-ray work, **17,** 222. The Inebriates' Acts, 1879–1899, **18,** 222. X-ray photographs as pictures, **19,** 131. Stereoscopic X-ray representation, **20,** 218. Twin X-ray representation and the reflecting stereoscope, **23,** 216. Proportional representation and the comparison of radiographs, **25,** 326. Radiographic estimation of simple enlargement by means of visible shadows, **28,** 226. Design for an elementary radiographic camera, **29,** 118. The right and wrong side of the X-ray picture, **30,** 227. Radiographic centroscope and the radiographic episcope, **32,** 202. Ten months in France with a field service X-ray outfit, **34,** 144.

**Crespi, A. J. H.,** Droitwich, **6,** 1. Bournemouth, **7,** 36. Inebriety, **7,** 244. Buxton, **9,** 167.

**Critchley, A. M.,** Ocular manifestations following encephalitis lethargica, **45,** 113. Clinical diagnosis of primary syphilis, **46,** 127.

**Critchley, M.,** The Parkinsonian syndrome, **41,** 25. Effects of lightning, **49,** 285.

**Cross, F. R.,** Glaucoma, **2,** 145. Intestinal obstruction, **3,** 34. Poisoning by duboisia, **4,** 127. Injurious effects of cocaine, **4,** 128. Phlyctenular ophthalmia, **4,** 132. Surgery of the upper eyelid, **4,** 186. Tumour in orbit, **5,** 189. **P.A.** Medical progress, **9,** 241. Asthenopia and ocular headache, **11,** 73. Eye symptoms of a case of extreme bradycardia, **17,** 113. Defects in the visual field, **29,** 33. **L.F.** Evolution of the sense of sight, **33,** 163. Early medical teaching in Bristol, **44,** 73. Lister, **44,** 113.

**Crouch, C. P.,** Membranous enteritis, **13,** 14. Granuloma of nose due to iodide of potassium, **21,** 231. Suggested treatment for functional aphonia, **25,** 214.

**Currie, A. S.,** Diphtheritic paralysis, **2,** 186.

**Cummins, S. L.,** Acquired resistance to tuberculosis, **40,** 41.

**Davidson, J. M.,** Radium and its physical and therapeutical properties, **28,** 1.

**Daukes, S. H.,** The Wellcome Museum of Medical Science, **46,** 236.

**Dacre, J.,** Aspiration of aorta, **3,** 189. Intubation of larynx, **6,** 73. Alcohol from a medical standpoint, **14,** 10. **P.A.** The family doctor, **23,** 289.

**Davey, J. G.,** Cremation, **3,** 81.

**Davies, D. S.,** Disinfection, **6,** 83. **P.A.** Modern preventive medicine, **18,** 289. Plague and diphtheria, **19,** 367. Variola nigra, **20,** 49. Public health, Bristol, **22,** 382 ; **24,** 137 ; **25,** 336. Voluntary notification of phthisis, **23,** 351. Prevention of disease, **24,** 193. Milk from a public health standpoint, **27,** 44. Small-pox in Bristol and neighbourhood, **27,** 97. Tuberculosis and natural selection, **30,** 135. Plague in Bristol, 1916, **35,** 16. Consumption in relation to public health, **37,** 211. With **Hall, I. W.,** Cerebro-spinal fever, **25,** 14. Typhoid fever and typhoid carriers, **26,** 39. **D.** and others, Encephalitis lethargica in Bristol, **37,** 25.

**Davies, N.,** Neuritis : its definition and successful treatment, **33,** 81.

**Davis, O. C. M.,** Progress of pharmacy and pharmaceutical chemistry, **23,** 120. Relationship between toxicity and chemical reactivity of certain benzene derivatives, **30,** 237. The 1914 British Pharmacopœia, **32,** 292. Clinical significance of adsorption phenomena, **37,** 205.

**Davy, H., and Blomfield, A. G.,** Locomotor ataxy with Charcot's joint disease, **10,** 249.

**Deakin, S.,** Pendulous fatty tumour of thigh, **3,** 51.

**Devine, H., L.F.** The reality of delusions, **45,** 19.

**Dobson, N. C.,** Abdominal section in perforating ulcer of stomach, **1,** 196. Intestinal obstruction, **3,** 28. Oral surgery, **7,** 108. **P.A.** Surgical aspects of carcinoma, **7,** 225.

**Dore, E.,** Radiation therapy in dermatology, **45,** 85.

**Dunbar, E. L. W.,** Diseases of children, **8,** 192. New theory and prophylaxis of puerperal eclampsia, **24,** 132.

**Eager, R., P.A.** Problems in psychology, **22,** 289.

**Edgeworth, F. H.,** Sequelæ of otitis media, **8,** 18, 87. Septic endocarditis, **9,** 89. Syringomyelia, **12,** 100. Blood poisoning in brothers, **13,** 107. Exophthalmic goitre, **14,** 41. Lumbago, **14,** 304. Diseases occurring in men who worked in a sewer, **16,** 33. Hysterical paroxysmal œdema, **16,** 206.

Achondroplasia, **17,** 27. Sub-acute bronchitis, **17,** 197. Initial signs of pleurisy, **21,** 36. Aneurysm of the ascending aorta, **22,** 23. Locomotor ataxy, **23,** 234. Acute yellow atrophy of the liver, **24,** 37. Alternating oxaluria and phosphaturia, **31,** 18. Treatment of chronic constipation, **31,** 40. Serous pleurisy due to pneumococci and tubercle bacilli, **31,** 146. Cerebral symptoms of lobar pneumonia in children, **31,** 308. Tachycardia in association with parenchymatous goitre, **32,** 144. **P.A.** The art of medicine in the age of Homer, **34,** 105. Causes of death in lobar pneumonia, **37,** 88. *See also* **Carter, T. M., Davies, D. S., Lees, E. L.**

**Elliott, C.,** Laryngeal phthisis, **2,** 260. Hyperpyrexia, **3,** 109. Intestinal intussusception, **4,** 177. Infantile scurvy or scurvy-rickets, **21,** 323.

**Elliott, F. P.,** Dietetic treatment of zymotic enteritis, **18,** 225. Presentation of the axilla at full term, **19,** 130. Compression of the aorta in treatment of post-partum hæmorrhage, **25,** 121, 310.

**Ellis, T. S.,** Surgical physiology of abdomen, **8,** 95.

**Evans, J. F.,** Cystic neuroma, **1,** 109. Muscular atrophy, **1,** 212. Unique case of attempted suicide, **3,** 124.

**Every-Clayton, L. E. V., P.A.** Medicine and surgery, **37,** 193.

**Fairbanks, W.,** Aspirator in retention of urine, **4,** 242.

**Faraker, E.,** Kala azar, **43,** 183.

**Faure, M.,** Treatment of tabetic ataxia by methodical exercises, **24,** 210. Mercurial treatment of tabes dorsalis, **27,** 312.

**Fawcett, E.,** Localization of the foramina at the base of the skull, **13,** 173. Explanation of alveolar cleft palate, **24,** 237. **L.F.** Development of the human skull, **28,** 97. With **Hadfield, G., and Phillips, P.,** Suprarenal virilism, **43,** 20.

**Fawn, G. F.,** Dental infections, **47,** 137.

**Fells, A.,** Pneumonia, **27,** 336.

**Fendick, R. G., and Parker, G.,** Case of extreme bradycardia, **17,** 113.

**Firth, J. L.,** Extra-peritoneal suppuration in perforating appendicitis, **14,** 36. Paralysis of respiratory centre from intracranial pressure, **15,** 139. Laparotomy, **16,** 229. Ligaturing the jugular veins in sigmoid-sinus thrombosis, **19,** 36. Torsion of the spermatic cord, **22,** 320. Fibroma of the abdominal wall, **24,** 304. Nephropexy, **31,** 220. **P.A.** Evolutionary history of renal surgery and temporal bone surgery, **41,** 1, 49. *See also* **Clarke, J. M.**

**Fisher, A. C.,** Pathology of erythema nodosum, **45,** 137.

**Fisher, A. G. T.**, Indications for manipulative surgery, **45,** 165.

**Fisher, T.**, Adherent pericardium in children, **12,** 93. Alcoholic disease of heart, **13,** 81. Fibroid degeneration of myocardium, **13,** 258. Rheumatic disease of cardiac muscle, **18,** 16. Sarcoma of intestine, **19,** 29. Displacement of abdominal viscera, **19,** 210. Ascites due to thrombosis of the hepatic veins, **20,** 209. Hæmatemesis associated with small white kidneys, **22,** 243. With **Neild, N.**, Congenital hypertrophic stenosis of pylorus, **22,** 123.

**Flemming, A. L.**, Ethyl chloride, **22,** 228. Filariasis with abscess, **26,** 139. Vomiting connected with anæsthesia, **27,** 40. Local analgesia in surgical operations, **32,** 193. Light ether anæsthesia, **40,** 88. **P.A.** Anæsthesia, **44,** 1. **L.F.** Partnership between anæsthesia and surgery, **48,** 233.

**Flemming, C. E. S.**, Achondroplasia, **17,** 21. Notes on three pathological specimens, **20,** 222. Lumbago, **24,** 110. Paroxysmal œdema of the lungs, **31,** 121. Medical education, **49,** 199.

**Fletcher, F. E.**, Cyanosis in the fatal broncho-pneumonia of influenza, **48,** 277.

**Fletcher, R.**, Some diseases bearing the names of saints, **30,** 295.

**Forrester-Brown, M.**, Treatment of infantile paralysis, **45,** 295.

**Fortescue-Brickdale, J. M.**, Collargol, **21,** 337. Blood-corpuscles in black-water fever, **22,** 130. Lac vinum infantum, **22,** 197. Scheme for infant milk depots, **22,** 132. Congenital dilatation of the ureters with hydronephrosis, **23,** 231. Anti-tuberculous vaccines and sera, **24,** 1. Proteins and protein metabolism, **24,** 318. Calmette's ophthalmic reaction in tuberculosis, **26,** 112. Heating of milk as it affects nutrition and health of the infant, **27,** 61. Sleep and modern hypnotics, **27,** 230. Hyoscine and mydriatic alkaloids, **28,** 317. Diagnosis in gastric cancer, **31,** 108. Ataxia, **31,** 235. Causes of variations in preparations of ergot, **31,** 279. Therapeutic notes, **31,** 279, 373 ; **32,** 88, 181, 249. Enteric group of diseases as seen in an isolation hospital in France, **34,** 72. Postgraduate study, **36,** 48.

**Fox, B. B.**, Masked epilepsy, **4,** 172. Hystero-epilepsy, **14,** 224.

**Fox, E. Long**, Compression of spinal cord by sarcomatous growths from soft membranes, **1,** 100. Spina bifida, **2,** 45. Chorea, **2,** 73. Intestinal obstruction, **3,** 17. Mangana water, **3,** 47. Enlargement of spleen, **3,** 145.

**Francis, F., L.F.** Relation between chemistry and medicine, **40,** 119. Colloidal state of matter, **45,** 177.

**Fraser, A. D.**, Endometriomata, **44,** 113. *See also* **Hall, I. W.**

**Fraser, F.**, Surgical treatment of abdominal conditions, **38,** 1. Surgical treatment of infections of the peritoneum, **40,** 29.

**Freeman, J.**, Administration of ether, **3**, 191. Chloroform or ether ? **14**, 137. The safety of the patient in anæsthesia, **27**, 238.

**Fry, E.**, Address to medical students, **11**, 267.

**Fry, J. D.**, *see* **Stock, W. S. V.**

**Fuller, J.**, Puerperal convulsions treated by venesection, **1**, 111.

**Fyffe, W. J., P.A.** Malarial poisoning, **1**, 143. Abscess of liver, **3**, 245. Reminiscences of an army surgeon, **7**, 145. Epidemic influenza, **8**, 147. Rötheln, **10**, 1. Double empyema, **11**, 233. Infectious diseases in a public school, **15**, 221.

**Garrett, J. H.**, Cheltenham, **11**, 241.

**Gibbs, A. N. G.**, Cerebral abscess, **2**, 121.

**Gillies, H. D.**, Plastic surgery, **43**, 122.

**Giraldi, J. J. J.**, Histology of the aortic wall in acute rheumatism, **46**, 145.

**Goodridge, H. F. A.**, Intestinal obstruction, **3**, 40. Aneurysm of aortic arch, **5**, 30.

**Goodwyn, H.**, Chronic appendicitis in its relation to dyspepsia, **35**, 18.

**Gordon, R. G.**, Coliuria in children, **32**, 286. Stammering as it occurred in the War, **36**, 143. Present position of psychotherapy, **38**, 11. Injuries to the head illustrating functions of the cortex, **40**, 139. Anterior poliomyelitis, **46**, 289. Child guidance, **47**, 126. Insomnia, **49**, 147.

**Gordon, W.**, Origin of gastric ulcer, **19**, 100. Value of blood-counts, **21**, 234. On digital percussion and the cardiac sign in carcinoma, **32**, 19. Reduction of disabilities from wounds in war, **34**, 1. Posture in physical examination of the heart, **42**, 224.

**Gordon-Watson, Sir C.**, Diverticulitis of the pelvic colon, **41**, 112.

**Goulden, C. B.**, Lachrymal obstruction, **26**, 143.

**Green, F. K.**, Testis of gout and of mumps, **1**, 227.

**Green-Armytage, V. B.**, Oriental medicine, **28**, 40.

**Greer, W. J.**, Spindle-celled sarcoma of the hard palate, **18**, 211. Craniotomy with implantation of egg-shell membrane (for Jacksonian epilepsy), **19**, 42. Surgical analgesia by spinal cocainization, **22**, 235.

**Grey, T. C.**, Cholecystotomy, **16**, 311.

**Griffiths, L. M.**, Shakespeare and the medical sciences, **5**, 225. Report on Medical Library, 1897–8 **16**, 369. The Hotwells, **20**, 1, 142, 193. Some things of general interest in Bristol Medical Library, **20**, 279. Growth of the Library of the Bristol Medico-Chirurgical Society, **20**, 376. The Medical Reading Society, Bristol, **25**, 222. Bristol Medical Library, **29**, 335. Queene Elizabethes Achademy, **37**, 1.

**Groves, E. W. H.**, Pelvic disease treated by laparotomy, **18,** 330. Diagnosis of malignant disease of the body of the uterus, **21,** 128. Tubal gestation producing severe hæmorrhage without rupture, **22,** 46. Advantage of enucleation of the tonsils over their removal by the guillotine, **23,** 32. Relation of the pelvic floor to pelvic displacements and pain in the female, **24,** 97. Spinal anæsthesia, **25,** 305. Trigeminal neuralgia treated by excision of Gasserian ganglion, **26,** 234. Tuberculosis of colon, **27,** 203. Excision of strictures of urethra, **28,** 325. Resection of the posterior spinal nerve roots, **29,** 105. Early diagnosis in acute intestinal obstruction, **30,** 37. Splenectomy in Banti's disease, **31,** 331. Apparatus for intratracheal anæsthesia, **31,** 347. The work of a military general hospital in Egypt, **34,** 11. Ununited fractures, **36,** 132. **L.F.** Repair of bone injuries, **38,** 142. Hospitals of Madrid, **44,** 229. Peri-arterial sympathectomy, **45,** 273. Borderland between surgery and gynæcology, **46,** 185. **P.A.** A surgical adventure : an autobiographical sketch, **50,** 1.

**Gubb, A. S.**, General anæsthesia by intra-spinal injections of cocaine, **22,** 231.

**Habershon, S. H.**, The secondary infections of pulmonary tuberculosis and their treatment by vaccines, **32,** 97.

**Hadfield, G.**, Renal efficiency, **39,** 28. Pathology of cervical erosion, **43,** 151. Central nervous system in Addisonian anæmia, **44,** 21. Pathology of coronary occlusion, **44,** 257. *See also* **Fawcett, E., White, E. B.**

**Hall, I. W.**, Clinical pathology, **24,** 121. Mixed infections, **31,** 227. Protective ferments, **31,** 304. Impurities of vended milks, **27,** 48. **P.A.** Contemporary medicine from the standpoint of pathology, **36,** 105. With **Fraser, A. D.,** Role of dilute acids in infection, **38,** 158. With **Smith, W. A.,** The value of some lactic acid ferments, **27,** 56. *See also* **Davies, D. S., Norgate, R. H.**

**Hanson, H. B.**, Women doctors with the army in the field, **33,** 88.

**Harper, J.**, Sub-diaphragmatic abscess, **2,** 184.

**Harrison, A. J.**, Factitious urticaria, **8,** 32. **P.A.** Comparative pathology, **12,** 273. **L.F.** Dermatitis from without and dermatitis from within, **24,** 325. Medical observations in Clifton Zoological Gardens, **31,** 318. With **Wills, W. K.,** Finsen light treatment of lupus and rodent ulcer, **21,** 23, 303.

**Harsant, W. H.**, Surgical notes, **1,** 82. Congenital syphilis, **1,** 83. Lupus vulgaris, **1,** 88. Strumous synovitis of elbow, **1,** 91. Suppuration in maxillary antrum, **1,** 93. Misplaced testicles, **1,** 96. Chancre on lip, **1,** 97. Congenital talipes, **1,** 97. Suppurating middle ear, **5,** 37. Surgery of thyroid, **6,** 265. Chloroform versus ether, **9,** 145. **P.A.** Medical Bristol in the eighteenth century, **17,** 297. Acute intestinal obstruction, **18,** 118.

**Harty, J. P. I.,** Electrical reactions in facial paralysis, **32,** 55. Experiences of an ear, nose and throat specialist at one of the bases, **34,** 39. Sinus phlebitis and thrombosis, **38,** 73. Disorders of deglutition, **40,** 195.

**Hayes, W. A.,** Myxœdema, **12,** 230.

**Hector, F. J.,** Premature infants, **49,** 301.

**Heimann, H. L.,** Sinoauricular block, **46,** 285.

**Herapath, C. E. K.,** Auricular fibrillation, **29,** 148. Treatment of Graves's disease, **47,** 193. *See also* **Coombs, C. F.**

**Hewer, T. F. R.,** Stokes-Adams syndrome produced by digitalis, **47,** 135. Changes in the spleen in acute pyogenic infections, **47,** 197.

**Hitchins, C. V.,** Weston-super-Mare, **7,** 198.

**Hughes, F. W. T., and Perry, C. B.,** Senile arterial changes in a child of seven weeks, **46,** 219.

**Iles, A. E.,** Intracranial tumours and papillœdema, **40,** 94. Lachrymal obstruction, **43,** 191. Prophylaxis of ophthalmia neonatorum, **44,** 125.

**Jackman, W. A.,** Parenchymatous goitre, **46,** 277.

**James, J. A.,** Case of lead encephalopathy, **27,** 124.

**Jenkins, F. G.,** Hæmaturia in pre-eruptive stage of measles, **48,** 275.

**Jessop, C. M.,** Latent rheumatism, **5,** 153.

**Joll, C. A., L.F.** Recent advances in diagnosis and treatment of cancer, **50,** 201.

**Jollye, F. W.,** Gouty peripheral neuritis, **6,** 28. Birth palsy, **7,** 87.

**Jones, E. J. T.,** Renal calculi, **21,** 245.

**Jones, H. L.,** Ionization or cataphoresis, **27,** 138.

**Jones, R. L.,** Vasomotor and ocular phenomena in rheumatoid arthritis, **20,** 328. Joint swellings simulating gout and rheumatism, **23,** 127.

**Jordan, L. R.,** Tuberculosis of tracheo-bronchial lymph nodes, **47,** 225.

**Karbeek, P. Q.,** Torquay, **11,** 93.

**Keall, W. P.,** Suturing of olecranon, **2,** 50.

**Keith, Sir Arthur,** Anthropology, old and new, **47,** 287.

**Kemm, N. E. F.,** Strangulated hernia, **48,** 151.

**Kent, A. F. S.,** Fatigue induced by labour, **35,** 47.

**Kesteven, W. B.,** Borderland of insanity, **3,** 97. Morbid appearance in brain of lioness, **3,** 246.

**King, P.**, Missing links in joint disease, **21,** 137. Static electricity, **24,** 233.

**Klein, E.**, Diagnosis of oriental or bubonic plague, **20,** 108.

**Kyle, H. G.**, Perforated typhoid ulcer, **21,** 30.

**Lace, F., P.A.** Developments in abdominal surgery, **36,** 1.

**Lane, Sir Arbuthnot W.**, Intestinal stasis, **33,** 1.

**Lansdown, F. P., P.A.** Progress of practical surgery, **4,** 217.

**Lansdown, R. G. P.**, Removal of bullets and other metallic foreign bodies, **33,** 157. **P.A.** Systematic examination of the abdomen, **36,** 33.

**Lawrence, A. E. Aust**, Treatment of incomplete abortion, **6,** 8. Post-partum complications, **8,** 73. Extra-uterine pregnancy, **10,** 73. Pigmentation in amenorrhœa, **12,** 107. **P.A.** Evils of marriage and pregnancy in women who do not possess sound pelvic organs, **14,** 289. Vascular caruncle of urethra, **18,** 23. Hæmorrhages of pregnancy, **19,** 196. With **Morton, C. A.**, Extra-uterine gestation, **11,** 29.

**Lawrie, J. M.**, Ovarian tumour in a young girl, **10,** 186.

**Lees, E. L., and Edgeworth, F. H.**, Hodgkin's disease, **30,** 150.

**Lilley, G. H.**, Emphysematous abscess of thigh from intestinal injury, **4,** 181.

**Lock, J. G.**, Tenby, **10,** 97.

**Lucas, J. J. S.**, Relationship of chorea and rheumatism, **22,** 239. Prognosis of pulmonary tuberculosis, **24,** 12.

**MacCormac, Sir W.**, Operation rooms, past and present, **16,** 301.

**Macdonald, P. W.**, Heart lesions and mental symptoms, **4,** 162. Some new hypnotics, **5,** 257.

**MacIlwaine, S. W.**, Senile nerve degeneration resembling general paralysis, **12,** 294.

**McKeag, R. H.**, Recent dental research, **47,** 143.

**Mackie, F. P.**, Diseases in Mesopotamia, **36,** 118.

**Maclean, Sir Ewen J.**, Multiple exostoses, **8,** 217. Quantitative estimation of the knee-jerk, **10,** 20. Ovarian tumour complicating pregnancy, labour and the puerperium, **23,** 40. Reminiscences and a forecast, **48,** 83.

**Mariette, E.**, Personal experience of pneumothorax, **30,** 189.

**Marshall, H.**, Rupture of aorta, **3,** 178.

**Meredith, J.**, Lymphadenosis, **3,** 255. Vomiting of pregnancy, **4,** 95. Menorrhagia and hygienics, **6,** 17, 102. Rötheln, **11,** 171.

**Mitchell, T.**, Causes of death in five cases in a colliery explosion, **15,** 235. Dislocation of the hand and carpus, **17,** 121.

**Moeller, M.**, Action of respirators, **2,** 136.

**Mole, H. F.**, Intubation in adults, **15,** 237. Ruptured tubal pregnancy, **21,** 39. Ruptured intestine, **25,** 38. Aural complications of influenza, **26,** 226.

**Moore, C. A.**, Urinary calculus, **31,** 126. Richter's strangulated hernia, **32,** 198. Surgical jaundice, **49,** 139.

**Morgan, C. Lloyd, L.F.** The conditioned response, **41,** 166. Personality : a review, **43,** 104.

**Morton, C. A.**, Rare form of spina bifida, **10,** 14. Multiple symmetrical joint lesions, **10,** 189. Duct cancer of the breast, **12,** 25. Cysts of unusual origin, **12,** 232. Operation for strangulated hernia, **14,** 23. Sudden dyspnœa, in goitre **14,** 213. Hysterical disease of both hips and both knees, **15,** 30. Hepatic and intestinal surgery, **15,** 317. Abdominal surgery, **17,** 203. Appendicitis, **18,** 320 ; **23,** 223, 360. Cancer of the breast, **20,** 305. Tuberculous disease of the hip-joint in childhood, **22,** 207. Preservation of limb in sarcoma, **24,** 308. Perforated gastric ulcer, **26,** 207. Stone in the urethra, **28,** 127. Surgical aspects of glycosuria and diabetic gangrene, **28,** 311. Cysts in the neck, **29,** 160. **P.A.** Present position of surgery of malignant growths, **29,** 289. Differential diagnosis of swelling in the breast, **30,** 121. Some unusual conditions found in operating for the radical cure of hernia, **32,** 12. Acute pancreatitis, **35,** 80. Gunshot injury of nerves, **36,** 55. Differential diagnosis of malignant disease of the cæcum from chronic and subacute appendicitis, **39,** 82. Obstruction due to gallstone, **41,** 126. *See also* **Clarke, J. M., Lawrence, A. E. A.**

**Munro, J. M. H.**, Is opsonic treatment useful in phthisis ? **26,** 97.

**Murgoci, H. B.**, Cancer arising in tissues within the duodenal loop, **44,** 195.

**Murray, J. A.**, Chemotherapeutic researches on cancer, **45,** 217.

**Muthu, C.**, Sanatorium treatment of pulmonary tuberculosis, **25,** 50. Early diagnosis of pulmonary tuberculosis, **26,** 131.

**Myers, C. S.**, Human improvability, **49,** 31.

**Neild, N.**, Treatment of hæmoptysis in tuberculosis, **26,** 124. Tea-poisoning, **29,** 312. Angina pectoris following sepsis, **44,** 263. *See also* **Fisher, T.**

**Neish, W. D.**, and **Tonkin, T. J.**, Leprosy in Jamaica, **22,** 1.

**Newnham, W. H. C.**, Vaginal hysterectomy for malignant disease, **15,** 24. Ruptured tubal pregnancy, **15,** 152. Hysterectomy for fibroid tumour of uterus, **17,** 8. Fibroid uterus, **32,** 37. **P.A.** Evolution of gynæcology, **32,** 257.

**Nierenstein, M.,** Modern chemotherapeutics, **36,** 151.

**Nixon, J. A.,** Muscular atrophy and sclerodermia, **21,** 328. Surgical aid in chronic ulcer of the stomach, **26,** 217. Thomas Dover, **27,** 31. Household syphilis and acquired syphilis in infants, **29,** 301. Neo-salvarsan in syphilis, **31,** 24. Splenectomy for splenomegaly, **31,** 325. Cotton-seed dermatitis and its cause, pediculoides ventricosus, **33,** 73. Famine-dropsy as a food-deficiency disease, **37,** 137. Progress in treatment of empyema, **38,** 82. **P.A.** The debt of medicine to the fine arts, **40,** 1. Rigid rest in rheumatic carditis, **43,** 96. Insulin in surgery, **43,** 199. Gastric and duodenal ulcer in infants, **45,** 72. **L.F.** Influence of food on the production and prevention of disease, **47,** 255. Silicosis—administrative and clinical, **48,** 253. Albuminuria in pregnancy, **49,** 229. Provincial medical journals, **49,** 312. *See also* **Bush, J. P., Davies, D. S.**

**Noble, J. I.,** Typhoid fever with co-existent bacillus coli infection, **47,** 323.

**Norgate, R. H.,** Effects of the war on mental conditions of the citizens of Bristol, **37,** 95. With **Hall, I. W.,** Institutional dysentery due to the Y. bacillus, **33,** 44.

**Norman, H. J.,** An old-time syphilidiator, **28,** 334.

**Ogston, A.,** Flat foot, **2,** 1.

**O'Leary, E. de L.,** Ethical aspects of physical research, **31,** 242.

**Ormerod, G. L.,** Pernicious anæmia, **49,** 61.

**Ormerod, H. L.,** Acute ulcerative endocarditis, **17,** 15. Care and treatment of mental defectives, **46,** 1.

**Orr-Ewing, H. J.,** Multiple serositis of unusual distribution, **38,** 90.

**Osler, Sir W.,** R. Fletcher, **30,** 289.

**Overton, R. E.,** Herpes zoster and varicella, **43,** 169.

**Owen, A. W.,** Infective character in thrombosis and embolism, **47,** 29.

**Parker, G.,** Branchial fistulæ, **11,** 89. Cases from an electrical department, **22,** 112. Causes of transient cerebral paralyses, **27,** 15. Biliary and intestinal sand, **28,** 112. Medical organization and growth of medical sciences in seventeenth century, **29,** 201. The Barber-Surgeons, **30,** 327. **P.A.** Foundation of the Bristol Medico-Chirurgical Society and the history of military medicine in England, **33,** 129. Erythræmia or splenic polycythæmia, **37,** 91. Uveo-parotitic paralysis, **43,** 73. Visit to Near East, **44,** 173. *See also* **Fendick, R. G.**

**Parsons, Sir J. H., L.F.** Metastatic inflammations of the eye, **27,** 1. Medicine and psychology, **39,** 1.

**Paterson, D. R.,** Peripheral neuritis, **8,** 244. Influence of the auricles on the percussion of the heart, **21,** 110.

**Penny, W. J.,** Rhinitis, **5,** 95. Patent urachus, **6,** 36. Affections of the tongue, **6,** 37. Surgical asthma, **6,** 41. Inflammation from cat-bite, **6,** 42. Constitutional syphilis, **6,** 45. Chancres of lip and gum, **6,** 46. Raynaud's disease, **6,** 48. Rheumatic orchitis, **6,** 49.

**Perry, C. B.,** Meningococcal septicæmia, **45,** 207. Congenital heart disease in school children, **48,** 41. *See also* **Hughes, F. W. T.**

**Phillips, P.,** *See* **Fawcett, E.**

**Philpots, E. P.,** Aachen, **3,** 104.

**Pickering, C. F.,** Fracture of femur, **3,** 48. Vesical calculi, **4,** 183. Acute inflammation of middle ear, **7,** 32. Septic thrombosis of lateral sinus, **9,** 155.

**Pinch, A. E. H.,** Therapeutic uses of radium, **41,** 97.

**Pinniger, W. J. H.,** Hæmorrhagic diseases of the new-born, **31,** 248.

**Pollard, G. S.,** Tuberculin test in cattle, **19,** 210.

**Posthuma, Mrs.,** Child guidance in New York, **47,** 111.

**Pountney, W. E.,** Needle in foot revealed by the X-rays, **14,** 238.

**Prichard, A.,** Rupture of aorta, **3,** 127. The medical certificate of cause of death, **6,** 153. Bristol Medical School, **10,** 264. One view of heredity, **11,** 91.

**Prichard, A. W.,** Fat embolism, **2,** 54. Aneurysm of orbit, **5,** 267; **8,** 115. Poisoning by rhus venenata, **9,** 22. Intussusception, **12,** 1. **P.A.** School surgery, **13,** 241. Fracture of frontal bone, **15,** 27. Fracture of patella, **17,** 104 Reminiscences of the Bristol Royal Infirmary, **18,** 193. Three unusual abdominal cases, **18,** 313. Artificial anus following herniotomy, **19,** 119. Subsequent history of a case of pylorectomy, **22,** 53. Mesenteric cyst, **22,** 332. The 3rd South Midland Field Ambulance, **31,** 159.

**Prichard, J. E.,** Addison's disease, **3,** 269.

**Priestley, W. D.,** Pathology of intra-uterine death, **6,** 55.

**Pridie, K. H.,** Mitral stenosis with auricular fibrillation, **45,** 125. The ventral position in the treatment of tuberculous spine, **50,** 261.

**Pringle, G. L. K.,** Irreducible intussusception, **17,** 322.

**Prowse, A. B.,** Retinoscopy, **1,** 200.

**Pullin, B. G.,** Treatment of warts, **5,** 269.

**Pye-Smith, P. H.,** Prognosis, **25,** 1.

**Pyke, H. D.**, Sugar tolerance tests, **45,** 129.

**Rattray, J. M.**, Postgraduate study, **28,** 193.

**Rayner, D. C.**, Tubal abortion, **20,** 323. **P.A.** Relation of gynæcology to glands of internal secretion, **48,** 1.

**Reinhardt, C.**, Styracol, **25,** 54.

**Renoirdre, Dr.**, Drainage continu par le gaz oxygène, **33,** 39.

**Robertson, D.,** and **Tasker, D. G. C.**, Rectal prolapse, **46,** 71.

**Rogers, B. M. H.**, Nitrous oxide before ether, **13,** 10. Isolation after diphtheria, **16,** 101. Idiopathic thrombosis of the portal and contributory veins, **17,** 107. Infantile scurvy, **21,** 318. Acute yellow atrophy of the liver in a child, **24,** 40. Narcolepsy, **25,** 87, 144. Physique of boys, **27,** 27. **P.A.** Medical aspect of Boswell's *Life of Johnson*, **28,** 289 ; **29,** 125. With **Bodman, J. H.**, Polyneuritis, **43,** 84.

**Rogers, H.**, Value of the chloride content of the cerebro-spinal fluid, **45,** 197. Necrosis of the myocardium, **48,** 35.

**Rogers, Sir L.**, The leprosy problem and its bearing on tuberculosis, **41,** 19.

**Rolfe, R.**, *see* **Stack, E. H. E.**

**Rose, F. H.**, Ethyl chloride, **20,** 53.

**Roué, W. B.**, Sudden death from asphyxia in phthisis, **2,** 181. Hydrocephalus, **4,** 102.

**Roxburgh, R., P.A.** Progress and practice, **16,** 285. Therapeutics of a sea voyage, **21,** 1. Round the world, **21,** 202. Lister, **30,** 1. Tuberculosis, **30,** 193.

**Rudge, C. K.**, The Bristol Medical Library, **30,** 344.

**Runnalls, H. B.**, Intestinal polypus, **3,** 181. Facial neuralgia, **3,** 249. Recuperative power of old age, **4,** 168.

**Russell, E.**, Adulteration of milk in Bristol, **27,** 47.

**Sargent, P.**, Surgery of the nervous system, **42,** 101.

**Savage, Sir G. H.**, Dreams and their significance, **30,** 156.

**Scarff, G. R.**, Tuberculous infection of the faucial and nasopharyngeal tonsils of children, **45,** 73. Familial multiple telangiectases of the skin and mucous membranes, **50,** 113.

**Scott, R. J. H.**, Dislocation of ulna and radius, **4,** 36.

**Semple, W. M.**, Graves's disease, **16,** 114. Method of copying sphygmograms, **16,** 312.

**Shaw, J. E.,** Ulcerative endocarditis, **1,** 114. Gastric ulcer, **2,** 42. Lateral sclerosis of spinal cord, **2,** 270. Retention and accumulation of fæces, **3,** 43. Hæmophilia with joint-lesions, **15,** 240. Points in lunacy practice, **15,** 301. Neuritic muscular atrophy (" peroneal type "), **17,** 319. Muscular atrophy due to lead, **19,** 117. With **Bush, J. P.,** Lesion of the cauda equina, **11,** 161. *See also* **Bush, J. P.**

**Sheen, A.,** Epidemic of tetanus, **1,** 173. Intestinal obstruction, **2,** 167. Diphtheria, **3,** 113. Pleural effusions, **11,** 14. Surgical aspects of Meckel's diverticulum, **19,** 310.

**Sheldon, T. S.,** Minute hæmorrhages in pia mater, **1,** 225. General paralysis of insane, **2,** 235.

**Shepherd, H. L.,** *see* **Statham, R. S. S.**

**Sheppard, A. L.,** Acquired angioma of the liver, **25,** 46.

**Shipway, F. E.,** Apparatus for intratracheal administration of ether, **31,** 341.

**Short, A. R.,** Iodoform and thyroidism, **28,** 122. End-results of operations on stomach and duodenum, **29,** 220. End-results of operations for internal derangements of the knee-joint, **30,** 52. End-results of cases operated on for gallstones, **31,** 34. Modern treatment of tuberculosis of the spine, **37,** 19. Enlarged prostate, **50,** 23. Cancer of the breast, **41,** 64. Diagnosis of dyspepsia, **42,** 88. **L.F.** Progress in surgical treatment, **46,** 243. Radium in the treatment of cancer, **47,** 82. Radium in exophthalmic goitre, **47,** 185.

**Short, L. J.,** The tuberculosis officer, **31,** 153. Diagnosis of tuberculosis, **35,** 1.

**Skerritt, E. M.,** Clinical evidence against contagiousness of phthisis, **1,** 48. Intestinal obstruction, **3,** 36. **P.A.** The teachings of failure, **10,** 229. With **Baron, B. J.,** Koch's treatment of tuberculosis, **8,** appendix.

**Slade-King, E. J.,** Ilfracombe, **8,** 257.

**Smith, F. S.,** Forensic medicine of blood-stains in the Tropics, **34,** 82.

**Smith, G. Munro,** Cardiograph in medicine, **1,** 71. After-treatment of scarlet fever, **2,** 263. Normal diet, **7,** 73. Pneumococci in the urine, **13,** 115. Spontaneous disappearance of a sarcomatous tumour, **18,** 30. **P.A.** The medical life, **20,** 289. Perforated duodenal ulcer, **21,** 218. Mesenteric thrombosis, **24,** 216. Rupture of middle meningeal artery, **26,** 58. " Vis medicatrix naturæ," **27,** 321. Herpes and brachial neuritis, **29,** 332. Bristol Medical Societies, **32,** 268.

**Smith, J. Greig,** Gastrostomy for malignant stricture of œsophagus, **1,** 122. Fatty tumour of thigh, **1,** 126. Scirrhus of jaw in child, **1,** 128. Meningocele, **1,** 129. Congenital deformity of right leg and left hand, **1,** 130. Ingrowing toe-nail, **2,** 99. Stricture of urethra, **2,** 154. Hæmophilia, **2,** 264. Intestinal obstruction, **3,** 1. Removal of uterine appendages, **4,** 1, 73. Calculus on foreign body in bladder, **4,** 38. Stone in bladder, **5,** 177. Hernia, **8,** 1. **P.A.** Modern medical journalism, **11,** 217. Extra-peritoneal closure of artificial anus and fæcal fistula, **13,** 1.

**Smith, L. S.,** Some complications of ruptured liver, **26,** 238. Furunculosis, **29,** 157.

**Smith, R. Shingleton,** Phthisical contagion, **1,** 1. Iodoform in phthisis, **1,** 218. Diphtheria, **2,** 61. Anterior poliomyelitis, **2,** 174. Intestinal obstruction, **3,** 20. Intrapulmonary injections, **3,** 164. Recent additions to dietary of sick, **4,** 55. Aneurysm of aorta, **4,** 233. Phthisis, **6,** 185. Phthisis and the germ theory, **6,** 225. Reports on medicine, **9,** 184, 284. Double spasmodic torticollis, **14,** 297. Scurvy as cause of hæmaturia in infancy and old age, **21,** 320. Examinations for life assurances, **23,** 11. Graves's disease, **23,** 317.

**Smith, W. A.,** *see* **Hall, I. W.**

**Smythe, H. J. D.,** Cervical erosion, **43,** 156. Normal puerperium, **46,** 59. Bacillus coli infection of female urinary tract, **50,** 243.

**Somervell, T. H.,** Mount Everest, **40,** 170.

**Souttar, H. S.,** Radium and its surgical applications, **46,** 19.

**Spencer, W. H.,** Peptonized food, **2,** 32. Gastric ulcer, **2,** 41. Thomsen's disease, **2,** 159. Pyrexia, **3,** 217.

**Spender, J. K.,** Medical posture, **1,** 184. Wounds of fingers, **3,** 73. Chloral, **7,** 1. Inflammation around cervical vertebræ, **11,** 1.

**Stack, E. H. E.,** Leontiasis ossea, **18,** 316. Cerebral-spinal meningitis, **19,** 44. Notes on operations on medical cases, **19,** 225, 326. Congenital deformity of nose ; injections of wax, **27,** 119. Gall-bladder cases, **30,** 49. Gunshot injuries to eyes, **33,** 198. **Stack** and others, Poisoning by mustard gas, **37,** 151.

**Statham, R. S. S.,** Sterility, **44,** 41. Dysmenorrhœa, **44,** 187. Albuminuria in pregnancy, **49,** 219. With **Shepherd, H. L.,** Extra-uterine pregnancy, **48,** 15.

**Stephens, G. A.,** Standard method of estimating blood-pressure, **50,** 121.

**Stephens, L.**, Suicidal perforation of stomach, **6**, 113. Congenital heart disease, **8**, 196.

**Stewart, J.**, Inebriety and so-called cures, **23**, 144.

**Stock, W. S. V.**, Anæsthetics, **46**, 197. With **Fry, J. D.**, A combined manometer and safety valve for intratracheal anæsthesia, **31**, 344.

**Stoddart, F. W.**, Bacterioscopic diagnosis of diphtheria, **13**, 18.

**Sturge, E.**, The misuse of medical charities, **22**, 273.

**Swain, J.**, Appendicitis, **12**, 9. Buccal glandular tumours, **13**, 94. Effects of Rontgen rays on calculi, **15**, 1. Abdominal operations, **16**, 211 ; **18**, 103 ; **19**, 18, 121 ; **20**, 33. Nephroptosis, **20**, 315. Conservative surgery of uterine appendages, **21**, 211. Abdominal tuberculosis, **22**, 31. Operation in myo-fibroma of uterus, **22**, 220. Surgical aspect of cholelithiasis, **23**, 208. Surgical diagnosis and treatment of vomiting in children, **24**, 116. Operation in carcinoma of pancreas, **26**, 45. **P.A.** History and practice of surgery in ancient and mediæval times, **27**, 289. Splenectomy, **28**, 223. Hypernephroma or mesothelioma of kidney, **31**, 213. British surgery in France, **34**, 128. Prevention of sepsis in war wounds by Carrel-Dakin method, **35**, 68. Torsion of great omentum, **37**, 202.

**Swayne, J. G.**, Placenta prævia, **1**, 166. Typhlitis, **4**, 108. Ought craniotomy to be abolished ? **5**, 1. The hour of delivery, **6**, 174. Accidental hæmorrhage, **7**, 170. Epithelioma of uterus, **8**, 119. Puerperal eclampsia, **9**, 1, 106. Forceps delivery, **10**, 153. Compression of umbilical cord during forceps delivery, **11**, 229. Ergot as an oxytocic, **12**, 225. Induced premature labour, **14**, 2, 126. Occipital presentations, **16**, 108.

**Swayne, W. C.**, Symphysiotomy, **17**, 11. Abdominal hysterectomy for fibroids of uterus, **18**, 123. Cæsarean section for obstruction of labour by pelvic tumour, **22**, 120. Cerebral lesions in pregnancy and parturition, **25**, 209. Operations on uterus and appendages during pregnancy, **30**, 31. **P.A.** Medical education, **30**, 316. Rectal diverticula as a causative factor in pelvic inflammation in women, **35**, 91. Prolapse and cystocele, **37**, 81.

**Symes, J. O.**, Secondary rashes in scarlet fever, **15**, 20. Bacteriology of some suppurations complicating pulmonary disease, **17**, 30. Antiseptic and disinfectant properties of soap, **17**, 193. Non-bacillary croup, **18**, 25. Pasteurization of milk, **18**, 275. On some urinary affections ; and urotropin, **20**, 27. Trypanosomiasis, **21**, 325. Æstivo-autumnal malarial fever, **22**, 126. School hygiene, **29**, 109. Heroin habit, **30**, 153. Renal efficiency, **39**, 25. Relationship of erythema nodosum to tuberculosis, **45**, 233. *See also* **Davies, D. S.**

**Symonds, H. P.**, Progress of surgery in last twenty-five years, **13,** 161.

**Symons, W. H.**, Statistics of consumption and other preventable diseases, **19,** 7.

**Taylor, A. L.**, Primary sarcoma of spleen, **46,** 121. Intestinal malformations and their clinical significance, **48,** 113. *See also* **Bodman, F. H.**

**Tayler, H. P.**, Ionization or cataphoresis, **27,** 132. Electricity in general practice, **27,** 145.

**Taylor, J.**, Tumour in brain, **2,** 128. X-ray history of a fracture, **18,** 214. X-rays in diagnosis of renal calculi, **20,** 44. X-ray therapeutics, **24,** 289.

**Taylor, J. W.**, Tuberculin and immunity, **30,** 338.

**Tasker, D. G. C.**, Renal disease, **43,** 161. *See also* **Robertson, D.**

**Temple, G. H.**, Treatment of eclampsia, **47,** 307.

**Thomas, J. L.**, Cure of hernia by Kocher's method, **16,** 307.

**Thomas, J. R.**, Abdominal section, **15,** 145.

**Thompson, G.**, Purulent sub-arachnitis, **1,** 107. Rupture of right auricle of heart, **2,** 48.

**Thomson, F. G.**, Referred cardiac pain, **27,** 193.

**Todd, A. T.**, Diabetes mellitus ; insulin, **40,** 182. Treatment of cancer, **44,** 144. Dyspepsia, **47,** 83.

**Tomkins, H. H.**, Spinal caries, **3,** 184.

**Tonkin, T. J.**, *see* **Neish, W. D.**

**Trevithick, E. G.**, A case resembling mucous colitis, **16,** 120.

**Turner, G.**, Surgery of malignant disease, **44,** 141.

**Tyrrell, W.**, Malvern, **9,** 267.

**Vachell, C. T.**, Cystic goitre, **5,** 264.

**Waldo, H.**, Aneurysm, **1,** 232. Raynaud's disease, **6,** 272. Factitious urticaria, **8,** 29. The paralysis of osteo-arthritis, **13,** 90. Baldness, **23,** 107. **P.A.** Syphilis, **25,** 289.

**Walker, C. H.**, **P.A.** Quackery in relation to diseases of the eye, **38,** 129.

**Wallace, J.**, Scarlet fever, **42,** 157.

**Walters, C. F.**, Autoplastic intra-medullary bone pegging for fractures, **32,** 139. A few suggestions in general surgery, **41,** 202. **P.A.** The duty of medicine to the race, **47,** 1.

**Warren, R.**, Appendicitis causing intestinal obstruction, **42,** 23.

**Waterhouse, R.,** Bromoform poisoning, **28,** 129. Aleucocythæmic leukæmia, **31,** 10. Cervical rib, **31,** 232.

**Watson-Williams, E.,** Hoarseness, **40,** 153. Labyrinthitis, **41,** 135. Comparative efficiency of local anæsthetics, **42,** 164. Operative treatment of meningitis, **43,** 91. Significance of vertigo, **44,** 119. Foreign bodies in the gullet, **46,** 109. Cancer of the tonsils, **47,** 78. Congenital abnormalities of the external ear, **48,** 273. Dysphagia with anæmia, **49,** 209.

**Watson-Williams, P.,** Morbus cœruleus, **6,** 183. Chronic pharyngitis, **7,** 189. Hay-fever, **10,** 84. Laryngeal tuberculosis, **9,** 27. Tumour of pons, **9,** 163. Syphilis and tuberculosis, **11,** 155. Egypt, **13,** 263. Paroxysmal tachycardia, **15,** 122. Pre-exanthematous sign of measles, **18,** 139. Hoarseness and loss of voice, **19,** 203. Diplococcal bronchitis, **20,** 135. On the probable rhythmical contraction of the bronchial muscular coat as a factor in pulmonary disease, **21,** 6. Throat as source of infection in acute rheumatism, **22,** 215. Why defective nasal respiration impedes growth and development, **24,** 17. Operations on nasal septum, **25,** 21. **L.F.** Suppurative disease of nose and ear, **26,** 1. Radical operation for chronic otitis media, **27,** 224. Direct laryngoscopy, bronchoscopy and œsophagoscopy, **30,** 11. **P.A.** Specialism and medical curriculum, **31,** 289. Treatment of laryngeal tuberculosis by tuberculin, **32,** 136. Pernasal operation for frontal sinus suppuration, **33,** 24. Sphenoidal explorations for meningococcal and other infections, **34,** 21. Operation for drainage of sphenoidal sinus, **34,** 157. Lymphosarcoma of the tonsil and palate, **37,** 51. Diagnosis of focal infection in the nasal sinuses, **42,** 121. History of *Bristol Medico-Chirurgical Journal,* **50,** 151.

**Weatherly, L. A.,** Malignant pustule, **2,** 124. Melancholia, **16,** 197. A plea for sanatoria for poorer consumptives, **18,** 305.

**Webb, W. H.,** Tonic contraction of lower extremities, **4,** 42.

**Wethered, F. J.,** Koch's treatment of tuberculosis, **8,** appendix.

**Whitby, C. J.,** Self-poisoning and its treatment, **14,** 307. Some thermal procedures, **18,** 1. The doctor as expert and pioneer, **24,** 222.

**White, E. B., and Hadfield, G.,** Pellagra, **44,** 31.

**White, W. H.,** Joint disease, **22,** 97.

**Whiteford, C. H.,** General anæsthesia, **18,** 217. Halsted's operation for cancer of breast, **18,** 336. Unreliability of microscope in diagnosis of malignant disease, **20,** 226. Ethyl chloride, **22,** 49. Epithelioma of tongue in young woman, **24,** 45. Tubal pregnancy, **24,** 126. Treatment of spreading peritonitis by drainage, **26,** 53.

**Wigmore, J.**, Medicine and its practitioners during earlier years of history of Bath, **31,** 193.

**Wigoder, S. B.**, Present position of radium therapy, **50,** 233.

**Wilkie, D. P. D.**, Acute intestinal obstruction, **47,** 97.

**Williams, C.**, Muscular atrophy, **5,** 103.

**Williams, E. C.**, Blackwater fever, **22,** 128. Infantilism, **22,** 247. Splenomegalic biliary cirrhosis, **24,** 42 ; **25,** 43, 88. Ataxia in a child, **29,** 163. Lymphatic leukæmia in a child, **32,** 147.

**Williams, P. W.**, *see* **Watson-Williams, P.**

**Williams, W. R.**, Myoma of uterus, **17,** 1. The varieties of uterine neoplasms, **17,** 314. Subungual exostosis, **22,** 17. Tumours and tubercle in monkeys, **25,** 149.

**Wills, W. K.**, Finsen light treatment of lupus, **21,** 119. Radiotherapy, **22,** 39. Acne vulgaris, **23,** 113. Lupus vulgaris, **25,** 127. Medical naval volunteer at Quebec, **26,** 327. Anomalous eruption showing bacillus morphologically corresponding with Klebs-Lœffler, **28,** 231. *See also* **Harrison, A. J.**

**Wohlmann, A. S.**, Significance of the human hand, **14,** 117. Bacillus of rheumatoid arthritis, **14,** 123.

**Wood, D.**, Vesical calculus in a urinary typhoid carrier, **37,** 148. Tetany following thyroid operations, **48,** 205.

**Wright, A. J. M.**, Tuberculosis of nose and pharynx, **30,** 45. Unsuccessful tonsil and adenoid operations, **40,** 84. Ear, nose and throat in influenza, **49,** 123. **L.F.** The problem of deafness, **49,** 256.

